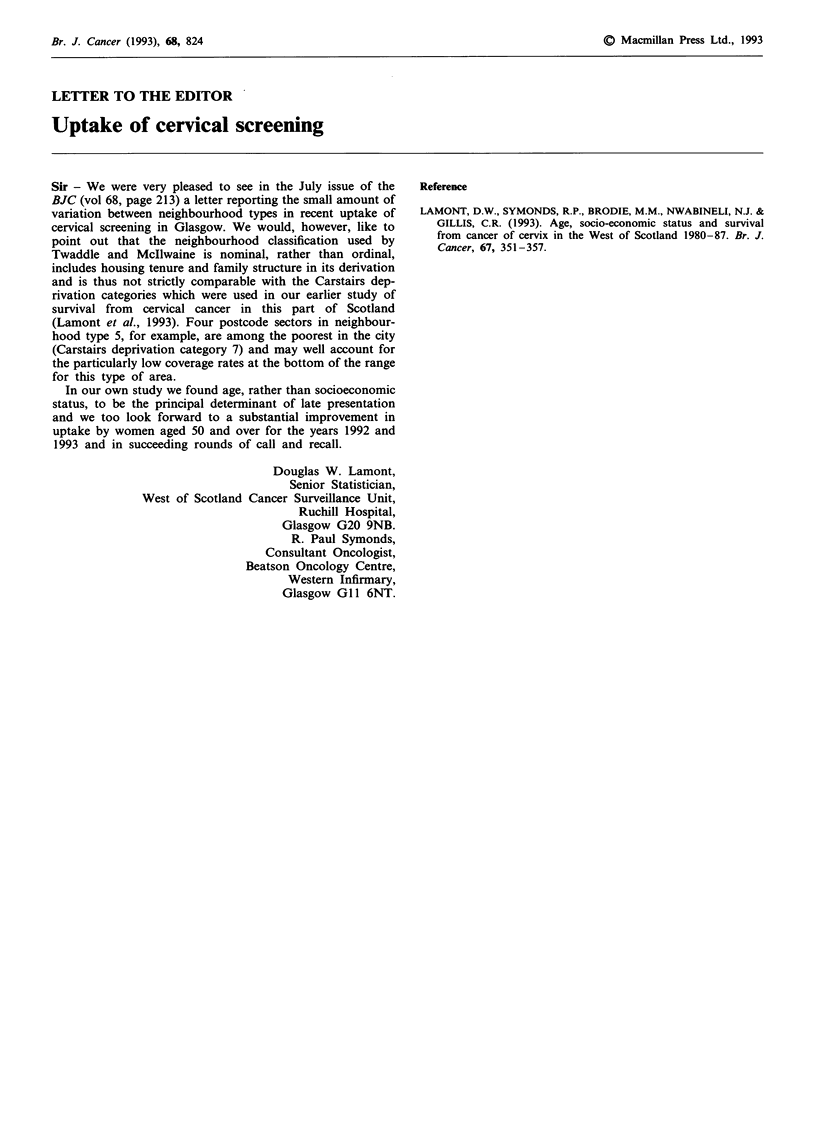# Uptake of cervical screening.

**DOI:** 10.1038/bjc.1993.435

**Published:** 1993-10

**Authors:** D. W. Lamont, R. P. Symonds


 © Macmillan Press Ltd., 1993

LETTER TO THE EDITOR

### Uptake of cervical screening

**Sir** – We were very pleased to see in the July issue of the *BJC* (vol 68, page 213) a letter reporting the small amount of variation between neighbourhood types in recent uptake of cervical screening in Glasgow. We would, however, like to point out that the neighbourhood classification used by Twaddle and McIlwaine is nominal, rather than ordinal, includes housing tenure and family structure in its derivation and is thus not strictly comparable with the Carstairs deprivation categories which were used in our earlier study of survival from cervical cancer in this part of Scotland (Lamont *et al.*, 1993). Four postcode sectors in neighbourhood type 5, for example, are among the poorest in the city (Carstairs deprivation category 7) and may well account for the particularly low coverage rates at the bottom of the range for this type of area.

In our own study we found age, rather than socioeconomic status, to be the principal determinant of late presentation and we too look forward to a substantial improvement in uptake by women aged 50 and over for the years 1992 and 1993 and in succeeding rounds of call and recall.

Douglas W. Lamont,
Senior Statistician,
West of Scotland Cancer Surveillance Unit,
Ruchill Hospital,
Glasgow G20 9NB.
R. Paul Symonds,
Consultant Oncologist,
Beatson Oncology Centre,
Western Infirmary,
Glasgow G11 6NT.

**Reference**

LAMONT, D.W., SYMONDS, R.P., BRODIE, M.M., NWABINELI, N.J. & GILLIS, C.R. (1993). Age, socio-economic status and survival from cancer of cervix in the West of Scotland 1980–87. *Br. J. Cancer*, **67**, 351–357.